# Triple P-Positive Parenting programs: the folly of basing social policy on underpowered flawed studies

**DOI:** 10.1186/1741-7015-11-11

**Published:** 2013-01-16

**Authors:** James C Coyne, Linda Kwakkenbos

**Affiliations:** 1 University of Pennsylvania, Philadelphia, PA 19104, USA; 2 University of Groningen, Groningen, The Netherlands; 3 Department of Psychiatry, McGill University, 1033 Pine Avenue West, Montreal, QC H3A 1A1, Canada; 4 Lady Davis Institute for Medical Research, Jewish General Hospital, 4333 Cote Ste Catherine Road, Montreal, QC H3T 1E4, Canada

**Keywords:** meta-analysis, publication bias, conflict of interest, dissemination, confirmatory bias

## Abstract

Wilson *et al*. provided a valuable systematic and meta-analytic review of the Triple P-Positive Parenting program in which they identified substantial problems in the quality of available evidence. Their review largely escaped unscathed after Sanders *et al*.'s critical commentary. However, both of these sources overlook the most serious problem with the Triple P literature, namely, the over-reliance on positive but substantially underpowered trials. Such trials are particularly susceptible to risks of bias and investigator manipulation of apparent results. We offer a justification for the criterion of no fewer than 35 participants in either the intervention or control group. Applying this criterion, 19 of the 23 trials identified by Wilson *et al*. were eliminated. A number of these trials were so small that it would be statistically improbable that they would detect an effect even if it were present. We argued that clinicians and policymakers implementing Triple P programs incorporate evaluations to ensure that goals are being met and resources are not being squandered.

Please see related articles http://www.biomedcentral.com/1741-7015/10/130 and http://www.biomedcentral.com/1741-7015/10/145

## Commentary

Wilson and colleagues [1] provided a great service to behavioral science and public policy by identifying serious limitations in the quality of evidence usually uncritically cited in support of the effectiveness of Triple P-Parenting programs. Using tools that can be readily applied by others, they showed the heavy reliance on self-referred, media-recruited two-parent families; a lack of comparisons between Triple P and alternative active treatments; assessment of outcomes with a battery of measures with little or no evidence of *a priori *designation of a primary outcome; biased reporting of findings in abstracts; and pervasive conflicts of interest with the authors of the bulk of the articles receiving royalties and other professional and financial rewards from the promotion of Triple P.

A defensive rejoinder from the promoters of Triple P, Sanders and colleagues [2], was disappointingly unresponsive, particularly in light of the extravagant claims currently being made for empirical support on Triple P websites [3,4] and in promotional material distributed around the world. Some of the risks of bias in the Triple P literature identified by Wilson and colleagues are indeed endemic to literature evaluating psychosocial interventions, but that does not excuse continuing promotion of Triple P without explicit acknowledgment of the limitations of the quantity and quality of available evidence. Sanders and colleagues' rejoinder underscores the problems of relying on developers and promoters of interventions to remain objective in evaluating their programs and in receiving criticism.

Yet, both Wilson and colleagues and the response from Sanders and colleagues overlook a fundamental weakness in the Triple P literature that amplifies other sources of bias. Namely, evidence consisting of studies with a high risk of bias is further limited by the preponderance of underpowered studies yielding positive results at a statistically improbable rate.

Wilson and colleagues noted a number of times in their review that many of the trials are small, but they do not dwell on how many, how small or with what implications. We have adopted the lower limit of 35 participants in the smallest group for inclusion of trials in meta-analyses [5]. The rationale is that any trial that is smaller than this does not have a 50% probability of detecting a moderate sized effect, even if it is present. Moreover, small trials are subject to publication bias in that if results are not claimed to be statistically significant, they will not get published with the justification that the trial was insufficiently powered to obtain a significant effect. On the other hand, when significant results are obtained, they are greeted with great enthusiasm precisely because the trials are so small. Small trials, when combined with flexible rules for deciding when to stop a trial (often based on a peek at the data), failure to specify primary outcomes ahead of time, and flexible rules for analyses, can usually be made to yield positive findings that will not replicate. Small studies are vulnerable to outliers and sampling error, and randomization does not necessarily equalize group differences that can prove crucial in determining results. Combining published small trials in a meta-analysis does not overcome these problems, because of publication bias and because of all or many of the trials sharing methodological and reporting problems [6].

What happens when we apply the exclusion criterion to Triple P trials of less than 35 participants in the smallest group? Looking at Table 2 in Wilson and colleagues' review, we see that 19 of 23 of the individual papers included in the meta-analyses are excluded. Figure 2 in the Wilson *et al*. review provides the forest plot of effect sizes for two of the key outcome measures reported in Triple P trials. Small trials account for the outlying strongest finding [7], but also the second-weakest finding [8], a likely sampling error from inclusion of small trials. Meta-analyses often attempt to control for the influence of small trials by introducing weights, but this strategy is inadequate when the bulk of the trials are small [9]. Again examining Figure 2, we see that even with the weights, such small trials still add up to over 76% of the contribution to the overall effect size. Of the four trials that are not underpowered [10-13], one [10] has a non-significant effect entered into the meta-analysis. In addition, the confidence interval for one of the positive, moderate-sized trials barely excludes zero (.06) [11].

Many of the trials evaluating Triple P were quite small, with eight trials having less than 20 participants (9 to 18) in the smallest group. This is grossly inadequate to achieve the benefits of randomization and such trials are extremely vulnerable to reclassification or loss to follow-up or missing data from one or two participants. Moreover, we are given no indication how the investigators settled on an intervention or control group this small. Certainly it could not have been decided on the basis of an *a priori *power analysis, raising concerns of data snooping [14] having occurred. The consistently positive findings reported in the abstracts of such small studies raise further suspicions that investigators have manipulated results by hypothesizing after the results are known (harking) [15], cherry-picking and other inappropriate strategies for handling and reporting data [16]. Such small trials are statistically quite unlikely to detect even a moderate-sized effect, and that so many nonetheless get significant findings attests to a publication bias or obligatory replication [17] being enforced at some points in the publication process.

Many communities and charities are proceeding with ambitious and costly implementations of Triple P-Parenting programs with the expectation that this will lead to the alleviation of social and public health problems associated with poor parenting. Wilson and colleagues highlighted the inadequacy of the existing clinical trial data. Adding to that the dominance of biased positive reporting of underpowered trials, it becomes incumbent upon clinicians and policymakers to adequately monitor the implementation of Triple P and evaluation of clinical outcomes to ensure that scarce resources are not squandered.

## Competing interests

The authors declare that they have no competing interests.

## Authors' contributions

Both authors contributed to the conceptualization, drafting and editing of the manuscript. Both authors read and approved the final manuscript.

## Pre-publication history

The pre-publication history for this paper can be accessed here:

http://www.biomedcentral.com/1741-7015/11/11/prepub
